# Feasibility of Human Amniotic Fluid Derived Stem Cells in Alleviation of Neuropathic Pain in Chronic Constrictive Injury Nerve Model

**DOI:** 10.1371/journal.pone.0159482

**Published:** 2016-07-21

**Authors:** Chien-Yi Chiang, Shih-An Liu, Meei-Ling Sheu, Fu-Chou Chen, Chun-Jung Chen, Hong-Lin Su, Hung-Chuan Pan

**Affiliations:** 1 Institute of Biomedical Sciences, National Chung-Hsing University, Taichung, Taiwan; 2 Institute of Life Sciences, National Chung-Hsing University, Taichung, Taiwan; 3 Department of Neurosurgery, Taichung Veterans General Hospital, Taichung, Taiwan; 4 Department of Medical Research, Taichung Veterans General Hospital, Taichung, Taiwan; 5 Faculty of Medicine, School of Medicine, National Yang-Ming University, Taipei, Taiwan; Boston Children’s Hospital and Harvard Medical School, UNITED STATES

## Abstract

**Purpose:**

The neurobehavior of neuropathic pain by chronic constriction injury (CCI) of sciatic nerve is very similar to that in humans, and it is accompanied by a profound local inflammation response. In this study, we assess the potentiality of human amniotic fluid derived mesenchymal stem cells (hAFMSCs) for alleviating the neuropathic pain in a chronic constriction nerve injury model.

**Methods and Methods:**

This neuropathic pain animal model was conducted by four 3–0 chromic gut ligatures loosely ligated around the left sciatic nerve in Sprague—Dawley rats. The intravenous administration of hAFMSCs with 5x10^5^ cells was conducted for three consecutive days.

**Results:**

The expression IL-1β, TNF-α and synaptophysin in dorsal root ganglion cell culture was remarkably attenuated when co-cultured with hAFMSCs. The significant decrease of PGP 9.5 in the skin after CCI was restored by administration of hAFMSCs. Remarkably increased expression of CD 68 and TNF-α and decreased S-100 and neurofilament expression in injured nerve were rescued by hAFMSCs administration. Increases in synaptophysin and TNF-α over the dorsal root ganglion were attenuated by hAFMSCs. Significant expression of TNF-α and OX-42 over the dorsal spinal cord was substantially attenuated by hAFMSCs. The increased amplitude of sensory evoked potential as well as expression of synaptophysin and TNF-α expression was alleviated by hAFMSCs. Human AFMSCs significantly improved the threshold of mechanical allodynia and thermal hyperalgesia as well as various parameters of CatWalk XT gait analysis.

**Conclusion:**

Human AFMSCs administration could alleviate the neuropathic pain demonstrated in histomorphological alteration and neurobehavior possibly through the modulation of the inflammatory response.

## Introduction

Neuropathic pain is defined as pain initiated or caused by a primary lesion or dysfunction of the nervous system [1, 2]. It is often caused by iatrogenic injury, traumatic injury, tumors compressing, chemotherapy drugs, diabetes or viral (HIV) diseases, and it frequently involves the peripheral nervous system [3]. Neuropathic pain is difficult to treat and generally poorly responsive to commonly employed therapies. Because of its very complex syndrome, neuropathic pain does not respond to traditional analgesics, such as anti-inflammatories antagonist and opiates. Currently, there are no clinical therapies for the neuropathic pain treatment that act in a complete and decisive way [4, 5].

Comparable to neurodegenerative diseases, neuropathic pain exhibits some responsiveness to stem cell therapy [6]. Most studies indicate that stem cells transplantation following spinal injury are capable of reducing allodynia and improve functional recovery [7]. In a variety of nervous injury models, the experimental data show that stem cells also possess of neuroprotective properties [8–10]. Mesenchymal stem cells (MSCs) have capacity to differentiate various tissue-specific lineages, and they demonstrate expansion potential, stability, and a self-renewing characteristic [11, 12]. The populations of stem cells involve some kinds of undifferentiated cells. Among them, MSCs are supposed to have the prime potential in pain research [6]. Recently, quantity of studies indicated that MSCs have immune-modulatory properties and anti-inflammatory effects [4]. Human MSCs are also found in the various tissues, such as adipose, skeletal muscle, umbilical cord, and the amniotic fluid [13–15]. The benefit of human MSCs is easy to be isolated from adult tissues, and their use is not restricted by ethical problems. Moreover, their powerful immunosuppressive abilities make them a viable candidate for transplantations [16–18]. Thus, the MSCs harbor substantial potential in pain therapy.

Amniotic fluid is known to contain multiple cell types which are derived from the developing fetus [19, 20]. It has been reported to be a fresh source for therapeutic transplantation of stem cells recently. Amniotic fluid—derived mesenchymal stem cells (AFMSCs) express features of both mesenchymal and neural stem cells. These two cell types have been used to treat various neurological disorders [21]. In our previous studies, the transplantation of amniotic fluid—derived mesenchymal stem cells facilitated peripheral nerve regeneration by the reason of the secretion of neurotrophic factors [22, 23]. In addition, AFMSCs have also been approved to facilitate nerve regeneration mainly through regulation of the inflammatory process [24, 25]. Due to the inherent characteristics of immunomodualtion, human AFMSC seems to be a potential source for nerve injury especially involved in an inflammatory response.

During the last decade, large volume of studies in various types of MSCs transplantation therapies has been reported in neuropathic pain. Local delivery of AFMSCs in injured nerve has been investigated, and the result demonstrated a significant improvement [23, 26]. In neuropathic pain models, MSCs have the ability to modulate pain behavior [27], although the underlying mechanism of alleviation on pain behavior requires further to be defined. Moreover, some series studies have already demonstrated that bone marrow MSCs were capable of reducing short-term pain-like behaviors and ameliorated pain-related molecular mechanisms in an intra-brain microinjection of neuropathic mice [28]. In another study, the experimental results also show long-lasting effects of mesenchymal stem cell through the systemic administration on alleviation in pain-like behaviors and decreased expression of inflammation associated bio-molecular markers in a spared nerve injury (SNI) mouse model [29].

Although the human AFMSCs belong to mesenchymal stem cells, the power of human AFMSCs in alleviating the neuropathic pain is still unknown. In the present study, we use a chronic constrictive injury rat model that mimicks the neuropathic pain to explore the potential of systemic intravenous administration of AFMSCs on neuropathic pain.

## Material and Methods

### Animal model

Sprague—Dawley rats (250–300 g) were housed four or five per cage and cared for in accordance with the guidelines recommended by Institutional Animal Care and Use Committee (IACUC). The protocol has been approved by Taichung Veterans General Hospital IACUU (La100869). During the experiments, food and water were feed ad libitum and animals were kept in an experimental environment at 20°C. These animals were placed in a room which with a disciplinary light/dark cycles of 12 h. The rats were cared by a veterinarian for at least 1 week before the commencement of experiments. The rats in experiment were anesthetized with 4% isoflurane for induction and maintenance with the dose of 1–2%. Chronic constriction injury model (CCI) were conducted with four 3–0 chromic gut ligatures loosely ligated around the left sciatic nerve 1cm above the trification as described previously [30]. After the surgery, 4–0 silk sutures were used for wound closure, and these animals were cared for at least 30 minutes before recovery. These animals were stochastically apportioned to three groups including sham operation, chronic constriction injury, and CCI with amniotic fluid—derived stem cells treatment.

The sham operation group consisted of the CCI surgery without four ligature ligations of the nerves. Groups of all experimental rats were assigned as follows:

(a) Sham-operated rat (n = 18) (Sham); (b) CCI rat treated with hAFMSCs (5x10^5^ cells once for three consecutive days) (CCI+hAFMSCs) (n = 18); and (c) CCI rat treated with PBS (CCI) (n = 18).

The animal pain-associated behaviors such as mechanical allodynia, thermal hyperalgesia, and Catwalk XT system were test for baseline (three days before injury), and weekly through the experiments. In addition, the evoked potential analysis was conducted one month after CCI.

### Preparation and culture of human AFMSCs

The methods in culturing hAFMSCs have already been described (1, 70). Non-adhering amniotic fluid cells in the supernatant medium after completeness fetal chromosome analysis on the fifth day were collected. The discarded AFMSCs were cultured in 5ml of β-minimum essential medium (β-MEM: Gibco-BRL) supplemented with 20% fetal bovine serum (FBS; Hycolone, Logan, UT, USA) and 4 ng/ml basic fibroblast growth factor (bFGF; R&D system, Minneapolis, MN, USA) in 25 cm flask and incubated at 37°C with 5% humidified CO_2_. The hAFMSCs were long-term passaged in culture, and 7–9 passages kept in culture before in vivo infusion. The hAFMSCs treatment group was intravenous injection with 5x10^5^ cells in 200μl PBS; the control groups were intravenous injection with 200μl PBS only. The cells were dissociated by 0.05% trypsin-EDTA (Gibco^®^) and wash two times (5 minutes for one washing time) with PBS, and immediately intravenous into animals. The time of the cells stored as a pellet or in suspension and washing until intravenous injection is approximately 10 to 15 minutes with the least delay possible. In the process of experiment, the cells kept sterile between transferring from culture to the animal in the sterile room. The written informed consents were obtained from all patients. This protocol had been approved by the Institutional Review Board of the Veterans General Hospital (950203/C06022).

### Isolation and culture of dorsal root ganglia cells (DRGs)

Dorsal root ganglia cells were obtained from the embryonic Sprague-Dawley rat in the embryonic days of 14 to 15. The rat was dissected according to the previous studies [31, 32]. The DRGs were incubated with 0.25% trypsin at 37°C for 15min and then dissociated. After dissociation, the cells were washed and re-suspended with Neurobasal medium containing 2% B27 (sigma, Inc.), 0.3% L-glutamine and 100ng/ml nerve growth factor. These cells were cultured in the dish at a density of 1x10^4^ cells per ml medium. Then, they were maintained and cultured in incubator at 37°C and 5% CO_2_. The cells were recognized by neuronal markers (βIII tubulin) before the experimental process.

### Nociceptive behavior

For behavior measurements, animals were performed on bilateral hind paws of all rats by researchers blind to managements. Mechanical allodynia was assessed using von Frey hair (Touch-Test Sensory Evaluator, North Coast Medical, Inc), as previously described in our group [33]. The method of allodynia assessment was modified by the previous procedure [34]. Each experimental rats were placed alone in a customized transparent acrylic chamber (20×20×20 cm) with a 5 mm thick acryl platform. In every trial of allodynia assessment, a serial various gram Von Frey hair to the bilateral hind paw five times at 5 seconds intervals or the moment that the hind paw was placed appropriately on the platform. If the rat which being testing did not withdraw the hind paw during five applications of a hair, the next larger hair was applied. The withdrawal threshold depended on the value (gram) of the hair which caused the hind paw withdrawal either four or five times out of the five applications. The other conventional assessment of pain behavior is thermal hyperalgesia. It was evaluated by hot-plate test (Technical & Scientific Equipment GmbH, TSE systems) according to the pervious procedure [35]. The time of paw withdrawal latency was recorded during the course of the rat touching the 52–54°C hotplate to the withdrawal of the paw. A protective proviso using a maximal cut-off of twenty seconds, was kept to prevent paw tissue injury.

### CatWalk automated quantitative gait analysis

The CatWalk XT gait analysis system was considered as a real-time and objective method compared with the traditional assessment such as Von Frey test in the various neuropathic pain models [36–38]. In brief, the CatWalk XT system comes with a high-speed digital camera with a sample rate of 100 frames per second. The video camera transforms each scene into a digital image. The digital images then are transferred to a computer through an Ethernet connection. The brightness of a pixel depends on the amount of light received from such an area by the camera. The Illuminated Footprint^™^ enables intensity difference to be detected between animals’ paws. The intensities vary from 0 to 225, and they are represented by different colors. The quantitative data such as duration of swing and stance phases, step sequence, print area, regularity index, and maximum contact maximum intensity were performed in all animals according to our previous report [33]

### Electrophysiology analysis

The somatosensory evoked potential was performed 1 month after nerve injury before the animal is sacrificed. The experimental rats were anesthetized and recorded by two electrodes. The electrode was threaded into the dural surface for detection of the somatosensory area (3 mm lateral and 2 mm posterior to the bregma). The reference electrode was placed over the maxillary area about 20mm from the active electrode. The stimulation electrode was placed on the sciatic nerve 1 cm proximal to injury area with intensity of 20mA and 20-2000Hz filtration. The data of conduction latency and evoked potential were presented as the ratio of right to left for the reducing the effect of anesthesia.

### Immunofluorescence analysis

All experimental animals were anesthetized and perfused with phosphate buffered saline (PBS), and followed by a fixative solution containing 4% paraformaldehyde. Brain, spinal cord, dorsal root ganglia, sciatic nerves and paw skin were obtained and immersed in 4% paraformaldehyde for 4–6 hours and transferred to 15% sucrose 6–8 hours. Then these tissues were transferred to the condition of 30% sucrose at 4°C overnight. The tissues were subsequently embedded in OCT compound (Tissue-Tek 4583; Sakura, Tokyo, Japan) and rapidly frozen before the immunohistochemistry analysis. Serial 8μm sections of the tissues were cut using a cryostat. After washing in PBS (5 minutes for three times), tissues was immersed for 30 min in blocking solution (1% BSA or 5% powdered skim milk). Primary antibodies were diluted in PBS and tissue slides were incubated at 4°C overnight in primary antibodies to PGP9.5 (Novus, 1:1000 dilution), anti-CD68 (Chemicon, 1:200 dilution), anti-S-100 (Neomarkers, 1:400 dilution), OX-42 (BD biosciences, 1:1000 dilution), anti-Synaptophysin (Abcam, 1:200 dilution), and anti-TNF-α (Abcam, 1:300 dilution). The fluorescent-labeled secondary antibodies (Jackson, 1:200 dilution) specific to the IgG species were used. Six samples in each immunohistochemistry staining were obtained for analysis. All the fluorescent imaging was performed with the same laser power and exposure time by the Olympus BX40 Research Microscope. The IHC images quantitatively analyzed by ImageJ and UN-SCAN-IT gel_TM_ (Gel & Graph Digitizing Software Version 6.1).

### Quantification of nerve fiber

In order to quantification of nerve fiber, three hindpaw skin sections were used per experimental rat and four fields of each section were analyzed. The number of immunoreactive intraepidermal nerve fiber was calculated following the labeled of fluorescently stain preterminal profiles. The standard of recorded was the fibers that were cross into the epidermis or branched in the epidermis. The experimenter was blind to all groups through the analysis.

### Western blot assay

The paw skin, the distal end of nerve tissue, dorsal root ganglion, somatosensory cortex, and hippocampus were obtained and proteins were extracted. Proteins were resolved by sodium dodecyl sulfate—poly-acrylamide gel electrophoresis and transferred onto a blotting membrane at the concentration of 50μg. The membranes were incubated with primary antibodies against PGP9.5 (Novus, 1:1000 dilution), S-100 (Neomarkers, 1:500 dilution), NF (Cellsignal, 1:1000 dilution), synaptophysin (Abcam, 1:500 dilution), TNF-α (Abcam, 1:1000 dilution), CD-68 (Chemicon, 1:500 dilution), and GAPDH (Santa Cruz Biotechnology, 1:1000 dilution) overnight at 4°C, after blocking. Then, the membranes were immersed with horseradish peroxidase—conjugated secondary antibody and developed using enhanced chemiluminescence western blotting reagents. Protein bands were analyzed by the ISI1000 image system (Alpha Innotech Corporation, CA, USA).

### Statistical analysis

These data were presented with mean ± standard errors. The Mann-Whitney U and repeated-measures ANOVA were used to compare the experimental results between control and experimental groups. The statistical analysis was managed by SPSS software version 12. If p-value was calculated less than 0.05, the result was considered significant.

## Results

### Effect of hAFMSCs on modulation of inflammatory response in primary dorsal root ganglia cells culture

**Fig 1:**
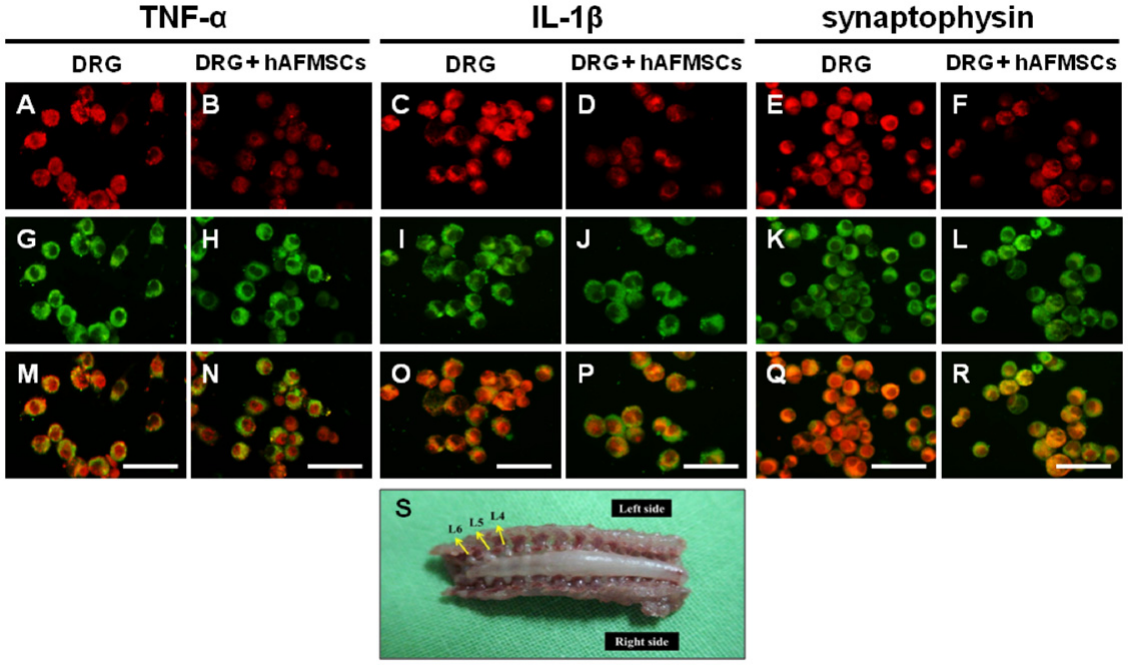
Illustration of the expression of TNF-α, IL-1β and synpatophysin over dorsal root ganglia cells primary culture either without or with co-cultured with hAFMSCs. (A, B) Expression of TNF-α in dorsal root ganglion without and with co-cultured with hAFMSCs. (C, D) Expression of IL-1β in dorsal root ganglion without and with co-cultured with hAFMSCs. (E, F) Expression of synaptophysin in dorsal root ganglion without and with co-cultured with hAFMSCs. (G, H, I, J, K, L) Expression of (βIII tubulin)in dorsal root ganglion cells in different groups. (M) Merged imaging of A and G, (N) Merged imaging of B and H, (O) Merged imaging of C and I, (P) Merged imaging of D and J, (Q) Merged imaging of E and K, (R) Merged imaging of F and L, (S) Illustration of dorsal root ganglion cells for culture. TNF-α, IL-1β, synpatophysin (red), andβIII tubulin (green): see text; Scale bar length = 100μm.

In our previous study, the expression of TNF-α, IL-1β and synpatophysin were upregulated after chronic constrictive injury [33]. To determine the effect of immunomodualtion, we performed the dorsal root ganglia cells primary culture (Fig 1S) and co-cultured with hAFMSCs. The results show that TNF-α, IL-1β and synpatophysin expression levels were remarkable assuaged by co-culture with hAFMSCs (Fig 1A–1R). These data suggested that hAFMSCs not only can suppress the secretion of inflammatory cytokines, such as TNF-α and IL-1β, but also alleviate the expression of synaptic vesicle protein.

### Effect of hAFMSCs on severity of histomorphology alteration in nervous system in chronic constrictive injury animal model

**Fig 2:**
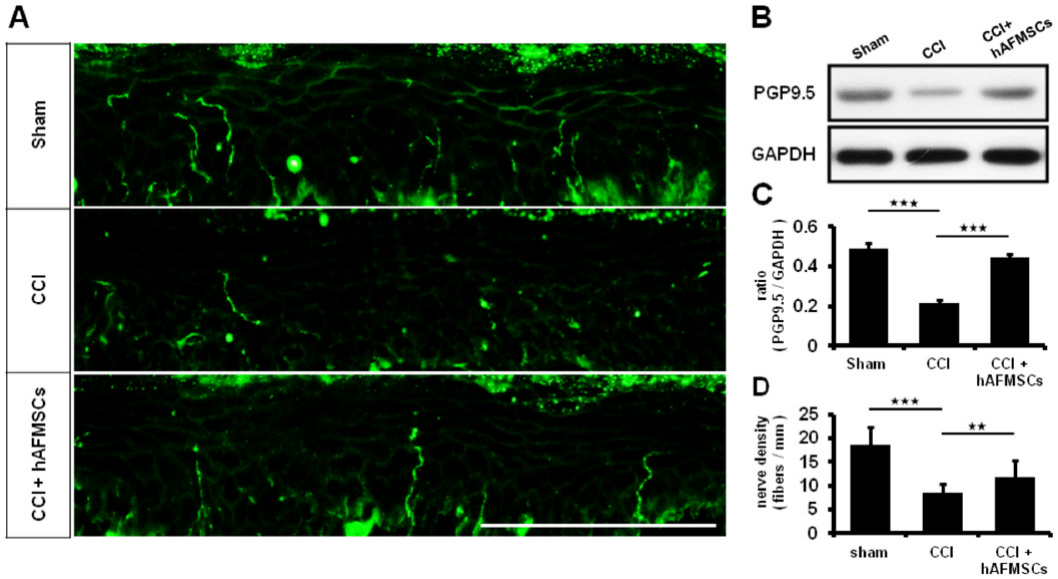
Expression of PGP 9.5 over the paw skin in different treatment groups. (A) Illustration of PGP9.5 over the hind-paw skin in different treatment group (B) Representative of western blot analysis in different treatment groups (C) The quantitative western blot analysis in PGP 9.5 in the paw skin (D) The quantitative analysis of nerve fiber density in epidermis. N = 6; *p < 0.05; **p < 0.01; ***p < 0.01; Scale bar length = 100μm.

Protein Gene Product 9.5 (PGP 9.5) was reported as ubiquitin C-terminal hydrolase 1 (UCHL-1). Decreased expression of PGP9.5 in axons of the epidermis and dermis was associated with neuropathic pain [39]. The highest expression of PGP9.5 in innervated skin was present in the sham group, and its expression was decreased in the CCI group. The administration of hAFMSCs returned the level of PGP 9.5 nearly to those in sham group (Fig 2A). The western blot analysis and nerve fiber density also showed the same trend (Fig 2B, 2C and 2D). Hence, CCI attenuated the expression of PGP9.5 in pawn skin, and this detrimental effect was restored by hAFMSCs.

**Fig 3:**
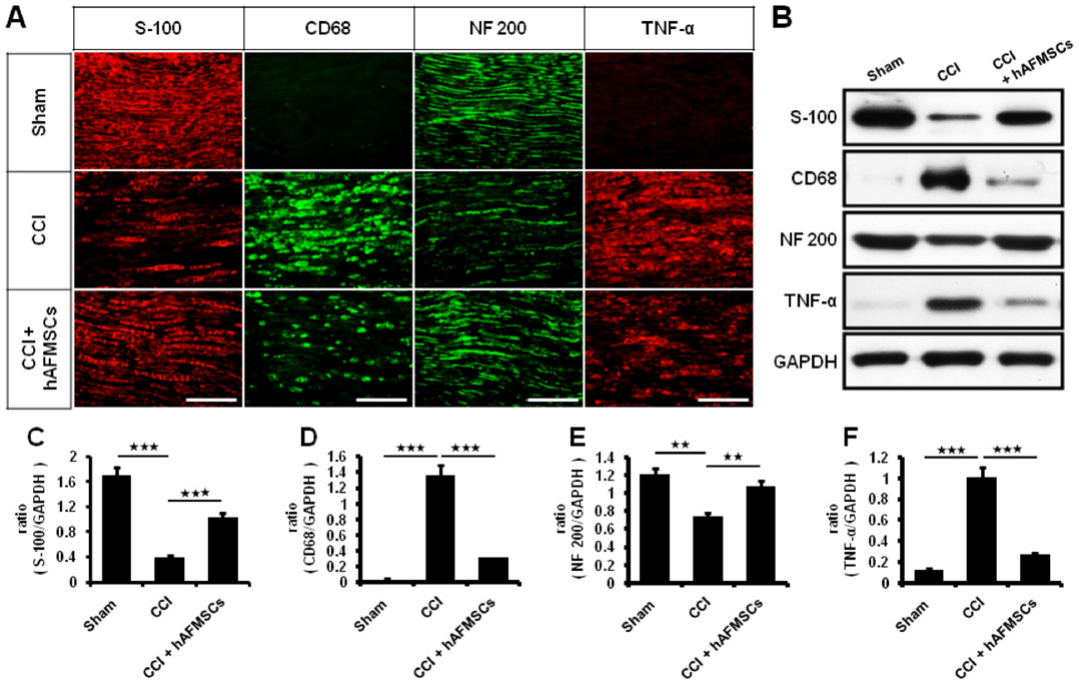
Expression of nerve regeneration and inflammatory response after human AFMSCs treatment. (A) Depiction of S-100, CD68, NF 200 (neurofilament)and TNF-α expression in the sciatic nerve in the different treatment, (B) Representative of western blot analysis of S-100, CD 68, NF 200, TNF-α, and GAPDH as a control), (C) Quantitative western analysis of S-100 in different groups, (D) Quantitative western analysis of CD 68 in different groups, (E) Quantitative western analysis of NF 200 in different groups, (F) Quantitative western analysis of TNF-α in different groups. *p < 0.05; **p < 0.01; ***p < 0.001. Bar length = 100μm.

Human mesenchymal stem cells are able to reduce pro-inflammatory gene and protein expression in neuropathic mice [28]. In order to investigate whether the hAFMSCs have the similar effects to hMSCs, we evaluated the expression of myelination and associated inflammatory cytokines in the distal end of nerve 7 days after CCI animals. The TNF-α expression level was significantly higher in the CCI group than in the sham group. Down-regulation of TNF-α was observed after administration of hAFMSCs (Fig 3A, 3B and 3F) (Table 1). The CD 68 deposition was also aggravated by CCI and then attenuated by hAFMSCs (Fig 3A, 3B and 3D) (Table 1). The reciprocal down-regulation of S-100 and neurofilament was noted after CCI and increased expression was observed after hAFMSCs (Fig 3A, 3B, 3C and 3E) (Table 1).

**Table 1:** Relative density of myelination and associated inflammatory cytokines in distal end of nerve subjected to different treatment.

	Sham	CCI	CCI+hAFMSCs	P value
S-100	2881.8±358.5	493.7±99.9	1063.1±23.9	<0.001
NF-200	5851.1±212.4	4347.1±718.9	6042.3±635.9	<0.05
CD 68	317.3±36.6	8449.4±763.8	2045.3±261.3	<0.001
TNF-α	62.6±42.8	2663.4±592.5	415.8±138.3	<0.001

Data was presented as mean±standard error.

S-100, NF-200, CD 68, TNF-α: see text.

Sham, CCI, CCI+hAFMSCs: see text.

**Fig 4:**
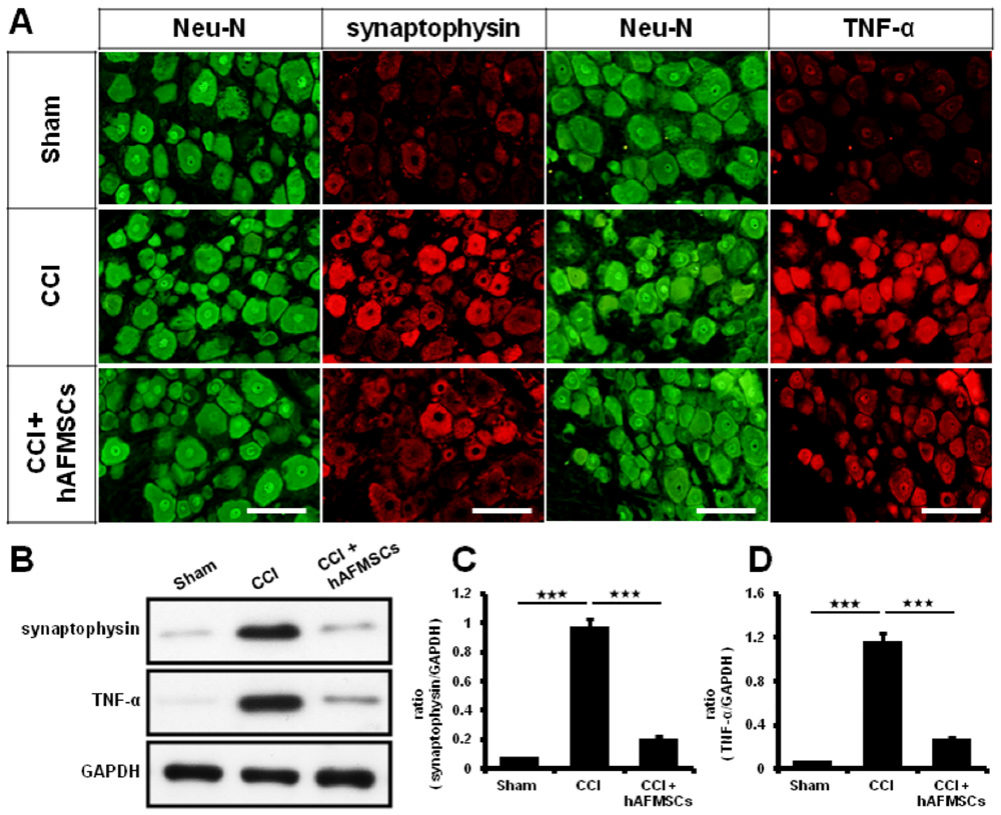
Expression of synaptophysin and TNF-α in dorsal root ganglion subjected to hAFMSCs treatment. (A) Representative of synaptophysin and TNF-α expression over the dorsal root ganglia in the different treatment, (B) Representative of Synaptophysin and TNF-α was detected by western blot analysis in different treatment, (C) Quantitative western blot analysis of synaptophysin in different treatment groups, (D) Quantitative western blot analysis of TNF-α in different treatment groups. N = 6 for western blot analysis, Scar bar length = 100μm. *p < 0.05; **p < 0.01; ***p < 0.001.

The expression of synaptophysin within the dorsal root ganglion tissues was highly correlated with severity of neuropathic pain [33]. In the present study, synaptophysin was highly expressed in the CCI group and significantly decreased when compared with the hAFMSCs treatment groups (p < 0.01) (Fig 4A, 4B and 4C) (Table 2). In the other hands, we also found that expression of TNF-α was approximate 2-fold higher in the CCI group than in the hAFMSCs treatment groups (Fig 4A, 4B and 4D) (Table 3).

**Table 2:** Relative density of synaptophysin over the various tissue in CCI subjected to different treatment.

	Sham	CCI	CCI+hAFMSCs	P value
Dorsal root ganglion	838.7±112.2	6022.5±788.4	2238.7±274.7	<0.001
Hippocampus	1072.1±282.4	5194.5±972.3	1864.8±658.2	<0.001
Somatosensory cortex	1234.5±247.8	4630.9±686.3	3007.6±253.8	<0.001

Data was presented as mean±standard error.

Sham, CCI, CCI+hAFMSCs: see text.

**Table 3:** Relative density of TNF-α over the various tissue in CCI subjected to different treatment.

	Sham	CCI	CCI+hAFMSCs	P value
Dorsal root ganglion	389.2±67.2	6884.9±749.3	3012.9±348.6	<0.001
Hippocampus	933.1±77.1	5612.1±235.9	3714.5±421.9	<0.001
Somatosensory cortex	1051±143.6	5750.9±891.3	3298.2±628.4	<0.001

Data was presented as mean±standard error.

Sham, CCI, CCI+hAFMSCs: see text.

**Fig 5:**
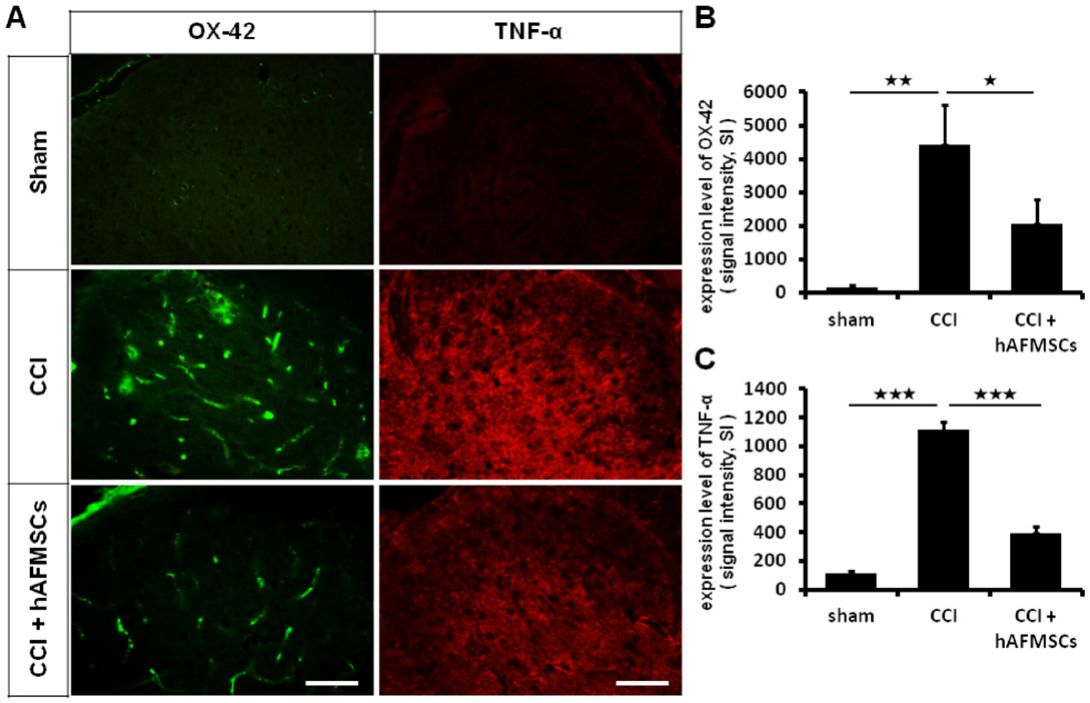
Expression of immune cells and associated inflammatory cytokines in dorsal spinal cord after hAFMSCs treatment. (A) Illustration of OX-42 and TNF-α expression over the spinal dorsal horn in the different treatment groups, (B) Quantitative analysis of OX-42 determined by the optic density in different treatment, (C) Quantitative analysis of TNF-α determined by optic density in the different treatment. Scar bar length = 100μm. N = 6 for analysis. *p < 0.05; **p < 0.01; ***p < 0.001.

To confirm whether the anti-nociceptive effect of hAFMSCs was due to a cellular phenotype alteration, we observed the specific glial marker and associated the inflammatory cytokines after the transplantation of hAFMSCs. On day 7 after chronic constriction injury, an increase in the number of Ox-42 cells was observed. This phenomenon was alleviated by the administration of hAFMSCs (Fig 5A and 5B). The level of TNF-α was also increased after injury and then attenuated by the hAFMSCs (Fig 5C).

**Fig 6:**
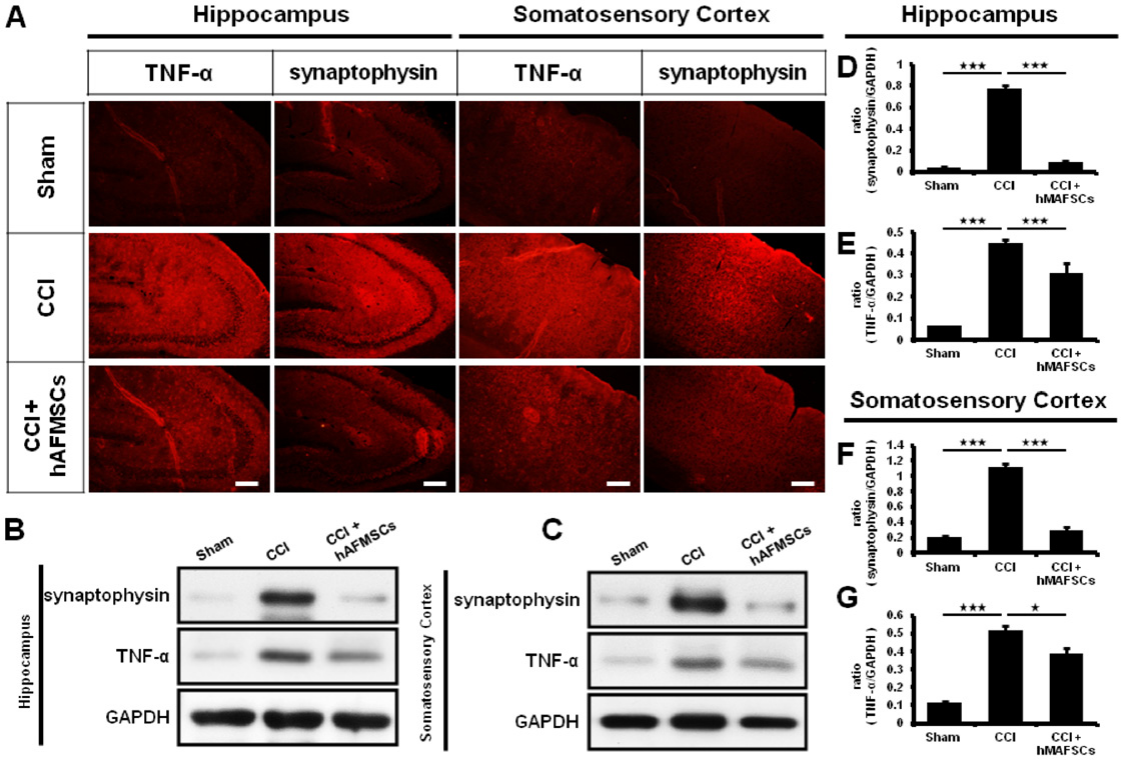
Expression of TNF-α and synaptophysin in hippocampus and somatosensory cortex after hAFMSCs treatment. (A) Depiction of synaptophysin and TNF-α expression over the hippocampus (CA3) and somatosensory cortex in different treatment groups, (B) Representative of western blot analysis of synaptophysin and TNF-α over the hippocampus in the different treatment, (C) Representative of western blot analysis of synaptophysin and TNF-α over the somatosensory cortex in the different treatment, (D) Quantitative western blot analysis of synaptophysin over the hippocampus in different treatment, (E) Quantitative western blot analysis of TNF-α over the hippocampus in different treatment, (F) Quantitative western blot analysis of synaptophysin over the somatosensory cortex in different treatment, (E) Quantitative western blot analysis of TNF-α over the somatosensory cortex in different treatment. N = 6 for western blot analysis, Scar bar length = 100μm,*p < 0.05; **p < 0.01; ***p < 0.001.

Moreover, synaptophysin and TNF-α expression within the hippocampus and somatosensory cortex mirror the brain sensory response to CCI injury [40]. In the present study, high levels of synaptophysin and TNF-α expression over the hippocampus were shown in the CCI group compared with the sham and hAFMSCs groups (Fig 6A, 6B, 6D and 6E) (Tables 2 and 3). Significantly high elevation of TNF-α was found in the somatosensory cortex between the CCI and sham groups. The administration of hAFMSCs exerted a decrease in the expression as compared to CCI group (Fig 6A, 6C, 6F and 6G).

**Fig 7:**
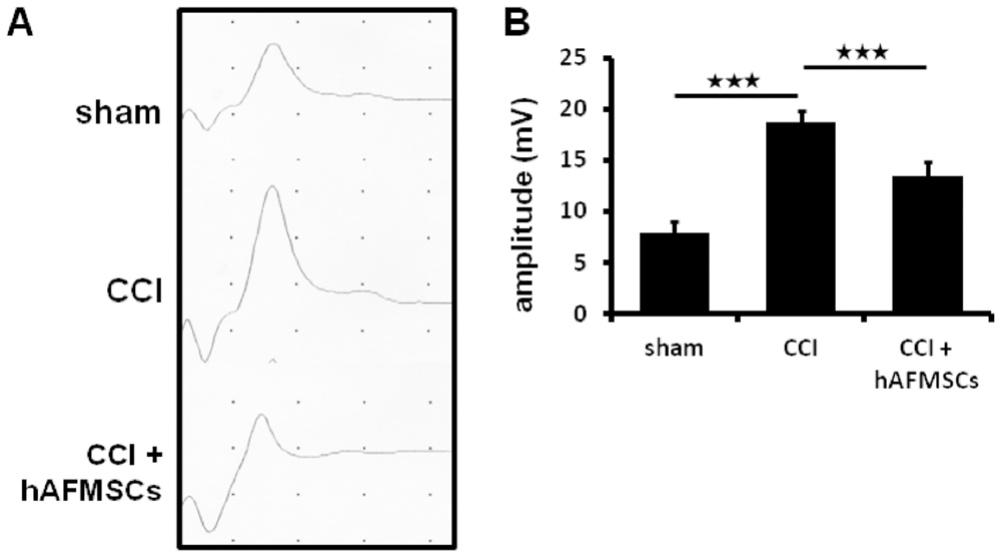
The alteration of somatosensory evoke potential after CCI and response after hAFMSCs treatment. (A) Plot of amplitude of somatosensory evoked potential in different treatment, (B) Quantitative analysis of the amplitude for the evoked potential in the somatosensory cortex in different treatment. N = 6 for analysis. *p < 0.05; **p < 0.01; ***p < 0.001.

In the previous analysis, the increased evoke somatosensory potential was highly correlated to severity of neuropathic pain [40]. Our results showed that the increased intensity of evoked potential was noted upon CCI injury. This increase was attenuated by the administration of hAFMSCs. The amplitude in the CCI group was more than 2-fold higher than that in the sham group (p < 0.01) and 1.4-fold higher than that in the hAFMSC-treated group (p < 0.01) (Fig 7A and 7B). These finding indicates that the chronic constriction injury in peripheral nerve was able to cause significant alteration in the electrophysiology and sensorimotor strip, and hAFMSC abolished this response.

### Effect of hAFMSCs on alleviating the mechanical allodynia and thermal hyperalgesia in pain behavior

**Fig 8:**
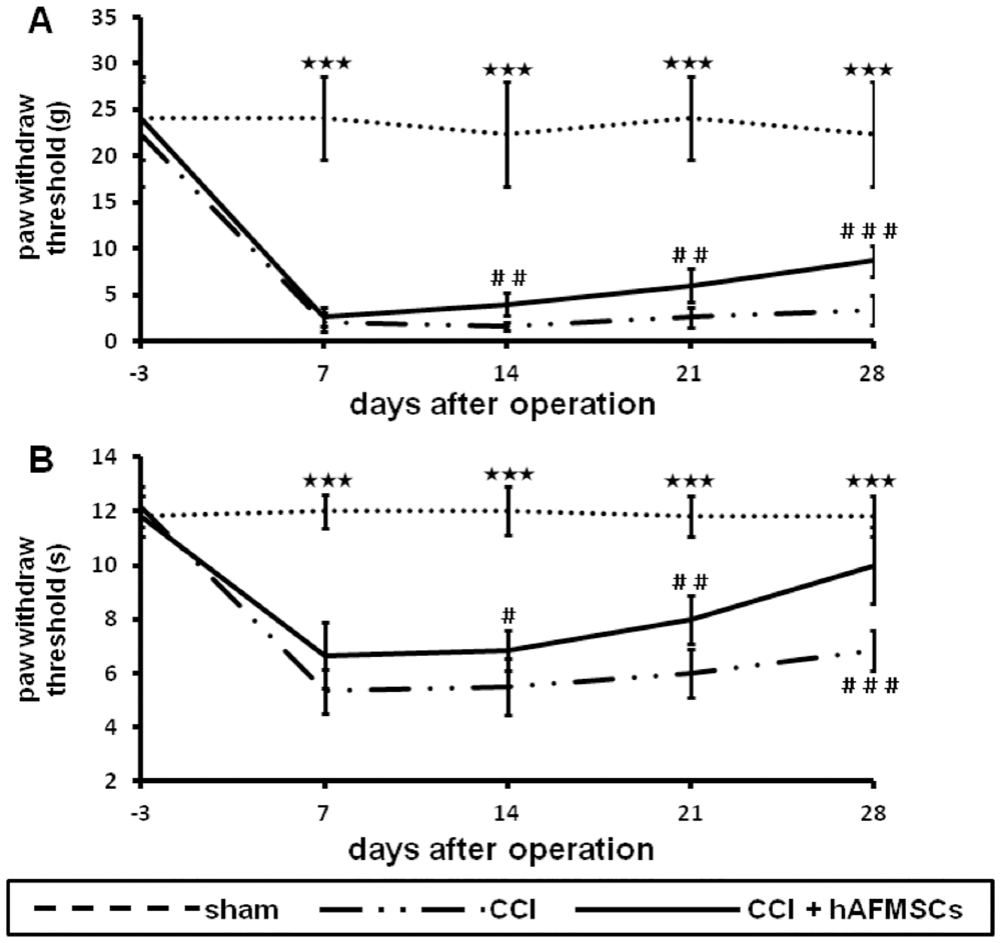
Plots of mechanical allodynia and thermal hyperalgesia related to different time frame in hAFMSCs treatment. (A) Depiction of mechanic allodynia related to different time frame in various treatments, (B) Illustration of thermal hyperalgesia related to the different frame in various treatments. N = 6, *p < 0.05; **p < 0.01; ***p < 0.001 for sham vs. CCI group; ^#^p < 0.05; ^##^p < 0.01; ^###^p<0.001 for CCI vs. CCI+AFS group.

Both the Von Frey and hot plate were usually used to assess mechanical allodynia and thermal hyperalgesia in neuropathic pain model. Chronic constriction injury caused a lasting persistent increase in mechanical allodynia and thermal sensitivity. The decreased threshold to mechanical allodynia after CCI plateaued 7 days postoperatively and persisted for at least 28 days after the operation. After hAFMSCs treatment, the progressively increased threshold of mechanical allodynia was noted at 14 days, and this trend was persistent up to 28 days after nerve injury (Fig 8A)

The most serious pain behavior assessed by thermal sensitivity was observed at 7 days after the surgery and prolong for 28 days. The thermal sensitivity effect was mild decreased 7 days after hAFMSCs treatment. The significant effect was noted at 14 days and all the way to day 28 (Fig 8B). Based on the previous result, hAFMSCs treatment could reduce the threshold of mechanical allodynia and thermal sensitivity, which reflect the ability in alleviating the neuropathic pain.

### Alteration of automatic CatWalk gait analysis subjected to hAFMSCs-treatment

**Fig 9:**
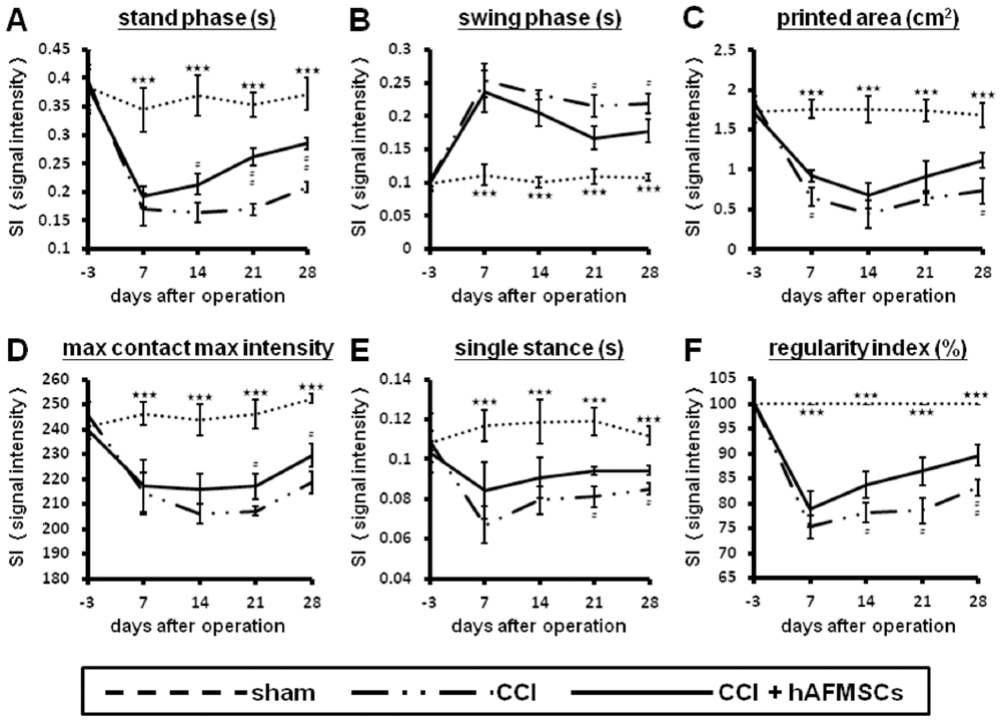
Illustration of various parameters of the CatWalk XT system related to various intensities of nerve damage at different time frames. (A) Illustration of stand phase in different treatment related to different time frames, (B) Illustration of swing phase in different treatment related to different time frames, (C) Illustration of printed area in different treatment related to different time frames, (D) Illustration of maximum contact maximum intensity in different treatment related to different time frames, (E) Illustration of single stance in different treatment related to different time frames, (F) Illustration of regularity index in different treatment related to different time frames. Printed area, Maximum contact maximum intensity, Stand phase, Swing phase, Single stance, and Regular index: see text. *p < 0.05; **p < 0.01; ***p < 0.001 for sham vs. CCI group;^#^p < 0.05; ^##^p < 0.01; ^###^p<0.001 for CCI vs CCI+AFS group.

CatWalk gait analysis can record subtle alternation in parameters such as intensity of printed area, maximum contact maximum intensity, stand phase, swing phase, single stance, and regularity index than mechanical allodynia and thermal sensitivity [33]. There existed significant differences of various recorded parameters between the CCI and sham groups as well as the CCI and the hAFMSCs-treatment group including print area, maximum contact maximum intensity, stand phase, swing phase, single stance, and regularity index (Fig 9A, 9B, 9C, 9D, 9E and 9F). Before the injury, the duration of the stance phase was 0.39 ± 0.06 seconds. In the CCI group, the duration of the stance phase was significantly reduced to 42.89% (p < 0.05) in one week after injury and 41.56% in two weeks after injury. Two weeks after injury, the duration of the stance phase for the hAFMSCs-treatment group was 55.69% (p < 0.05) lower than that the group before injury and 57.83% lower than the sham group (p < 0.05) (Fig 9A). Two weeks after injury, the swing phase increased to 232.79% of the preoperative value (p < 0.001). In the hAFMSCs-treatment group, the swing phase recovered to 19.98% of the CCI group (Fig 9B). Print area is the surface area of the complete print. In the hAFMSCs-treatment group, the print area recovered to 17.69% of the CCI group (p < 0.001) (Fig 9C). The preoperative intensity of the left hind paw (injury site) was 240.96 ± 3.46 arbitrary units. Two weeks after CCI, the max contact max intensity was significantly reduced to 83.77% of the preoperative value (p < 0.001) (Fig 9D).

In the CatWalk XT system, the regularity index and single stance were high correlated to inter-limb coordination, which reflected the pathological brain dysfunction [33]. This alteration of regularity index and single stance reached the valley at 7 days either in CCI and hAFMSCs groups. In the hAFMSCs groups, either RI or single stance led to significant improvement as compared to CCI from the time points of 7 days to 28 days (Fig 9E and 9F).

## Discussion

In the present study, we demonstrated for the first time that systemic administration of hAFMSCs through tail vein was capable to alleviate neuropathic pain behavior through the reducing the expression of pain-associated pro-inflammatory cytokines. In single intravenous stem cells administration, most of the intravenous injection of hMSCs was deposited in the lung, and this caused the stem cell treatment difficult when they were intended for other organs [41]. In our previous study, we modified the intravenous administration method and demonstrated that continuous intravenous administration of hAFMSCs was mostly deposited in lung in the beginning and then recruited to the injured nerve to facilitate nerve regeneration [42]. However, it has been also report that the human amniotic fluid—derived stem cells are low tumorigenic probability [15], there is no tumor formation or asthenia over any organs in animals during our experiment.

In this study, we used our previous method of intravenous admission of hAFMSCs and found that there exerted the significant relief of neuropathic pain, and these effects were mostly through the modulation of inflammatory response.

In the last decade, the therapeutic influence of MSCs has been an abundant evolvement and the recent study explored the potential of MSCs therapy, including stimulation of resident stem or progenitor cells, remodeling of the extracellular matrix, and stimulation of neogenetic blood vessel formation, morphological characteristics, neural markers, and electrophysiological properties [3, 43–45]. Furthermore, some studies also indicated that tissue repair was not merely through the direct effect from cell replacement and mainly by the way of paracrine function or releasing protective trophic factors [3, 28, 46, 47]. In this study, we cannot demonstrate the integration of the hAFMSCs into the injured nervous system but through the immunomodualtion of hAFMCs similar to the effect of MSCs. We expect that there is an accurate method for tracking hAFMSCs in vivo homing or integration after intravenous injection in our further investigations.

The initiation and maintenance of neuropathic pain was not only through the neuronal intrinsic pathways but also components of the peripheral immune system [48]. After tissue injury, the inflammation was generated by the activation of innate immune cells and glia cells. The immune-active substances which from the immune cells, including cytokines, neurotrophic factors and chemokines, which activate local actions and can lead to a serious generalized immune response and sensory nerve sensitization [49, 50]. There are several experiments demonstrating the pleiotropic effects of MSCs on the immune system by secreting molecules factors and regulating cells, which includes dendritic cells, B cells, and T cells [51, 52]. In accordance with these properties, MSCs can suppress the improper activation of T lymphocytes and create an appropriate environment during tissue repair, thus conduce to the maintenance of immune homeostasis [53, 54]. In our study, the decreased neuropathic pain by hAFMCs was through the modulation of inflammatory response, and associated immune cells distributions were in line with the reports of MSCs.

MSCs are reported to have the properties of immunosuppression that can take advantage of successful autologous and heterologous transplantations without demanding pharmacological control [16]. In the spared nerve injury (SNI) model, intra-brain microinjection of human bone marrow-derived mesenchymal stem cells (hMSCs) were capable of reduced pain behaviors, GFAP-positive cells activation and cytokines expression, such as IL-1β [28]. The pain relief response was very similar to systemic administration of neural stem cells (NSCs) mainly through the expression of Fos, SP, and CGRP in the spinal cord [55]. The adipose-derived mesenchymal stem cells were another source for MCSs and harbored similar properties to bone marrow-derived mesenchymal stem cells and neural stem cells, which also had the power in pain relief [3]. In the rat models, human umbilical cord blood (hUCB-MSCs) and amniotic epithelial stem cells (hAESCs) were also reported in alleviating of mechanical allodynia after spinal cord injury in rats. The postulated mechanism was due to decreased NMDA receptor subunit expression in the central nervous system and reduced activation of microglia [47]. In our study, hAFMSCs administration reduced the inflammatory response from the skin to brain, which paralleled the results from the MSCs.

The markers of hAFMSCs have been analyzed and indicating express mesenchymal and neuronal stem cell markers (CD29, CD44, CD73, CD90 and CD105), but negative for hematopoietic stem cell markers (CD45, CD34 and CD133) [56]. In addition, AFMSCs also present immunosuppressive properties just like those of other source of MSCs [57]. MSCs express low degree of MHC class I and no class II molecules and lacking co-stimulatory molecules such as CD86, CD40 [58]. The properties of immunomodualtion in hAFMSCs were similar to those in MSCs. Our results of immunomodualtion characteristic of hAFMSCs also showed the same trend with those of MSCs.

Dorsal root ganglia cells are located in the intervertebral foramen of spinal cord and involved the sensory neuron innervating the peripheral tissues. It was reported that these cells can be used as a proper model for the experiment of nerve regeneration [59]. Human mesenchymal stem cells can promote neurogenesis in primary cultures of peripheral nerves, and they also express protein such as neuron-specific protein tubulin β-3 (Tuj1) for promoting neurite growth [60, 61]. Furthermore, the DRG cell co-cultured with differentiated adipose-derived stem cells also showed the regeneration properties [62]. For the determination of the immunomodualtion of hAFMSCs, we performed the co-culture of hAFMSCs and DRGs. The results indicated that inflammatory cytokines such as TNF-α, IL-1β protiens, and synpatophysin expression levels were more assuaged by co-culture with hAFMSCs. These data strongly suggested that hAFMSCs not only can suppress the secretion of inflammatory cytokines but also alleviate the expression of synaptic vesicle protein.

Although MSCs were trapped in the lungs, experimental evidence has accumulated to indicate that MSCs are able to homing to injured tissues after systemic delivery [63, 64]. Studies pointed out that inflammation directs migration of transplanted MSCs to the injury site [65–67]. These phenomena were consistent with the data as we previous showing that hAFMSCs can be recruited by SDF-1-α in injury model [42]. In the present study, we found that escalated expression of inflammatory cytokine was distributed to the nervous system which suggests that the systemic administration of hAFMSCs may be involved in immune-modulations.

There was significant alteration in inflammatory response from skin to the spinal cord when subjected to nerve injury [33]. Decreased expression of PGP9.5 in axons of the epidermis and dermis was associated with neuropathic pain [39]. High expression of TNF-α, and CD68 cells deposits were demonstrated in nerve injury [33]. The expression of TNF-α and synaptophysin in dorsal root ganglion reflect the status of inflammatory reaction [33, 68]. The expression of TNF-α and immune cell deposit in dorsal spinal cord was in line with severity of inflammatory response [33]. From our histomorphological analysis and associated protein determination, the local inflammation responses from the skin to dorsal spinal cord were significantly attenuated by hAFMSCs administration. Although these results indicated that hAFMSCs may be involved in immune-modulations, we still cannot explain that the injected cells are preventing denervation after CCI injury directly. In our previous study [33], S100-positive cells and NF200-positive nerves alterations were associated with severity of nerve damage. However, these results may offer an interesting thinking, nerve denervation may be prevent by decreasing inflammation by hMAFSCs immune-modulations after CCI injury.

Some experimental evidence suggests that peripheral sensory dysfunction caused by injury or inflammation should be due to not only the cellular and biochemical reaction happening but also to functional and anatomical alteration in the cerebral somatosensory cortex [69–71]. The increased evoked potential amplitude were correlated with the intensity of nerve damage, and these increases paralleled with substantial expression of synaptophysin and decreased expression of TNF-α within the brain somatosensory system [33, 72]. Our results showed the attenuation of evoked potential and expression of TNF-α and synaptophysin in somatosensory system by hAFMSCs administration.

The CatWalk XT system is a comprehensive tool for automated gait analysis in animals for determination of animal behavior change [73–75]. In the previous study, the CatWalk XT system not merely assessed the trend in intensity of neuropathic pain, but compare the delicate alterations in neurobehavior associated with histomorphological changes [33]. The regularity index and single stance indicated the dysfunction in the somatosensory system and also correlated with the intensity of memory and associated inflammatory cytokines expression [33, 74, 76–78]. In this study, the remarkable improvement in Catwalk parameters by hAFMSCs especially of single stance and regularity index further conferment the alteration in somatosensory alteration demonstrated by evoked potential and associated inflammatory response.

In the CCI model, the pain-like behavioral alters such as mechanical and thermal hyperalgesia, were observed to develop within a week. These changes of neuropathic pain persisted for at least 7 weeks after the surgery, and recovered to normal behavioral from 2 to 4 months [30, 79, 80]. In order to eliminate the self-recovering, we only observed 28 days for behavior analysis, until sacrifice. In the experimental group, the tendency of behavior was a trend back towards sham levels. Although we only observe animal behavior to 28 days after surgery, but we still expect animal behavior can approach a full recovery.

## Conclusions

The systemic administration of hAFMSCs is able to comprehensively regress neuropathic pain in a rat CCI model. Human AFMSCs alleviate the neuropathic pain demonstrated from the histomorphological alteration and associated inflammatory response from the skin to the brain somatosensory systems. The neurobehavior assessed by CatWalk and Von-Frey and thermal plate was also in line with the histomorphological alteration and associated inflammatory response. The application of hAFMSCs in alleviating the neuropathic pain seemed promising.
